# Effect of Season and Parity on Reproduction Performance of Iberian Sows Bred with Duroc Semen

**DOI:** 10.3390/ani11113275

**Published:** 2021-11-16

**Authors:** Javier Piñán, Beatriz Alegre, Roy N. Kirkwood, Cristina Soriano-Úbeda, Magdalena Maj, Juan Carlos Domínguez, Rodrigo Manjarín, Felipe Martínez-Pastor

**Affiliations:** 1 Institute of Animal Health and Cattle Development (INDEGSAL), Universidad de León, 24071 León, Spain; javierpinan@gmail.com (J.P.); ctibag@unileon.es (B.A.); c.soriano.ubeda@unileon.es (C.S.-Ú.); jcdomt@unileon.es (J.C.D.); felipe.martinez@unileon.es (F.M.-P.); 2 Department of Animal Medicine, Surgery and Anatomy (Animal Medicine and Surgery), Universidad de León, 24071 León, Spain; 3 School of Animal and Veterinary Sciences, University of Adelaide, Roseworthy, SA 5317, Australia; roy.kirkwood@adelaide.edu.au; 4 Department of Biological Sciences, California Polytechnic State University, One Grand Ave., Bldg. 10, San Luis Obispo, CA 93407, USA; mmaj@calpoly.edu; 5 Animal Science Department, California Polytechnic State University, One Grand Ave., Bldg. 10, San Luis Obispo, CA 93407, USA; 6 Department of Molecular Biology (Cell Biology), Universidad de León, 24071 León, Spain

**Keywords:** Iberian, gilt, sow, Duroc boar, artificial insemination, fertility, prolificacy, parity, seasonality, cosinor

## Abstract

**Simple Summary:**

The Iberian pig is appreciated for the organoleptic and nutritional characteristics of its meat. Traditional breeding is extensive, but many farms perform intensive breeding for increased productivity. In these cases, Duroc boars are the source of semen for artificial insemination (AI). There is little information on this kind of breeding; therefore, we analyzed the records from a farm near Mérida (Spain). Our main interest was the effect of parity (number of previous farrowings) and season on the reproductive results of the farm. Sows between 2 and 4 previous farrowings showed the best fertility and prolificacy. Additionally, the results were higher for AI performed in autumn and winter, with a drop in reproductive performance earlier in the year than for other breeds, which usually decline by summer–early autumn. Increasing day length in the spring combined with higher temperatures in late spring–summer could lower performance during these periods. These results could help improve the management of Iberian sow farms.

**Abstract:**

The Iberian pig is an autochthonous breed from the Iberian Peninsula highly valued for its meat. The sows are often bred as Iberian × Duroc crossings for increased efficiency. Since sow parity and season affect the reproductive performance, we evaluated two-year records from a commercial farrow-to-finish farm (live, stillborn, and mummified piglets after artificial insemination, AI). A total of 1293 Iberian sows were inseminated with semen from 57 boars (3024 AI). The effects of parity (gilts, 1, 2–4, 5–10, and >10 farrowings) and season were analyzed by linear mixed-effects models (LME). The data were fitted to cosinor models to investigate seasonal effects within parity groups. The effects of maximum daily temperature (MDT) and day length change (DLC) during spermatogenesis, pre-AI, and post-AI periods were analyzed with LME. The 2–4 group was the optimal one for parity. A seasonal effect was evident between spring–summer (lower fertility/prolificacy) and autumn–winter (higher). Cosinor showed that the seasonal drop in reproductive performance occurs earlier in Iberian sows than in other breeds, more evident in gilts. MDT negatively affected performance in all periods and DLC in spermatogenesis and pre-AI. These results are relevant for the improvement of Iberian sows’ intensive farming.

## 1. Introduction

The Iberian pig is a highly valued breed from the Iberian Peninsula, recognized for its organoleptic and nutritional characteristics [1,2]. Whereas traditional rearing is extensive or semi-extensive, demand has led to intensive management of the animals [1]. These are typical of Iberian × Duroc crossings, adding the meat characteristics of the Iberian breed to the advantages of Duroc genetics (precocity, leanness, and higher performance). The use of artificial insemination (AI) with liquid semen doses from Duroc boars enables increased efficiency [3] and allows for the planning of crosses according to the desired product by modulating the proportion of Iberian genetics [1]. Despite the increasing importance of this production system for the breed, there are little data on the reproductive efficiency of Iberian sows or their crosses after AI with semen from Duroc boars. Current data are limited to reports from producers, which lack systematic research on this topic, and we are not aware of publications on the reproductive performance of these crosses.

The reproductive performance of pigs depends on many factors. Due to its importance for swine production, it has been extensively analyzed for many breeds and situations [4,5,6,7,8]. Parity is a relevant factor, with the number of farrowings influencing sows’ fertility [9,10]. There are important differences between gilts (nulliparous) and multiparous sows [4]. Gilts present physiological features that result in overall lower fertility and prolificacy [11,12,13]. Moreover, low-parity sows could be more sensitive to environmental stress [14,15].

The effect of parity is complicated by the influence of management and breeding systems [8], and whereas gilts can show a good prolificacy, their performance can vary greatly depending on the breed and management [16]. Similarly, older sows, with many farrowings, are more prone to reduced reproductive performance. Sows in parities between two and five are considered the most productive, showing an optimal prolificacy [5,17].

Another critical factor for pig reproduction is the environment, mainly influenced by the season and local climatology. Whereas modern farms can efficiently control the temperature (especially in dry climates [18]) and photoperiod, these factors still significantly impact pig production. However, the seasonal effect is more important when using breeds with a lower level of genetic selection or in latitudes with a higher thermal or humidity variation [11,19,20]. Indeed, we detected a seasonal effect in studies using commercial breeds (Landrace, Large White, and their crosses) [21,22,23]. However, when studying the sperm cryopreservation of the autochthonous breed, Gochu Asturcelta (Northern Spain), the seasonal effect was small and, interestingly, it reflected on a lower freezability in winter [19]. In this regard, the Iberian pig is peculiar in many aspects [1,2], and this could affect parity and season effects on its AI performance differently compared with other breeds. Iberian boar semen could be more sensitive to specific conditions during extension and storage [24], and a role for environmental factors can be speculated. Therefore, a characterization of Iberian × Duroc production farms is relevant for improving reproductive efficiency.

In this study, we analyzed records from an Iberian sow farm using Duroc semen for AI, with the objective of evaluating the impact of both sow parity and season on their fertility and prolificacy. Due to the interest and peculiarities of the Iberian breed, the study aimed to find the similarities and differences with more common commercial breeds in order to optimize the management of the Iberian × Duroc breeding system.

## 2. Materials and Methods

### 2.1. Animals and Farm Procedures for Semen Collection and AI

The study was performed by evaluating records of sow performance to AI in an Iberian sow farm (farrow-to-finish) near Valverde de Mérida (38° 54′ N, 6° 13′ W, 267 m MSL, Badajoz, Spain) for two consecutive years. Iberian sows (n = 1293), between 8 and 98 months old, were housed in individual gestation stalls after AI and until confirmed pregnant at 35 d after mating. Pregnant sows were housed in pens with 37–38 sows/pen until they were moved to individual farrowing crates from 1 week before farrowing until weaning at 28 d. The temperature was maintained between 18 °C and 24 °C using coolers, and the photoperiod was maintained at a minimum of 12 h using artificial lighting. For the first post-weaning estrus detection, sows had boar contact for 5–10 min/d for 15 d from weaning.

Semen doses were obtained from 57 Duroc boars 12–30 months of age located on a farm near Mérida (38° 55′ N, 6° 20′ W, 224 m MSL, Badajoz, Spain), following routine protocols for AI centers. The boars were kept under controlled conditions with a regulated temperature between 18 °C and 23 °C, and at least 12 h of daily light. These boars were subjected to semen collection once weekly. Semen was collected using the hand-gloved technique and immediately diluted in MR-A^®^ commercial extender (Kubus, Madrid, Spain) and packaged in collapsible squeeze bottles (2 × 10^9^ spermatozoa, 45 mL). Semen doses were shipped to the farms at 15 °C and used within 48 h of collection. Sows were inseminated at estrus detection and then at 24 h intervals while exhibiting estrus. The parameters measured were fertility (farrowing rate) and the total piglets born, live piglets born, stillborn, and mummified piglets.

To analyze the effect of parity, we classified the animals as gilts (never farrowed), parity 1, parity 2–4, parity 5–10, and parity > 10, as previously described [5]. The complete records ranged from 2016 to 2018, including 3024 AI performed with semen from the 57 Duroc boars in the 1293 Iberian sows. For sow parity, the distribution of AI was 256 gilts, 178 with one farrowing, 974 in the 2–4 group, 1525 in the 5–10 group, and 91 sows with >10 farrowings.

### 2.2. Meteorological Data

The study area shows a Csa climate according to the Köppen classification (i.e., Mediterranean hot summer), characterized by an annual average temperature of 17 °C and 368 mm of total precipitation. Rainfall occurs during winter, whereas summers are hot and dry. Meteorological variables were obtained from AEMET (Spanish State Meteorological Agency, http://www.aemet.es; accessed on 12 September 2020) using the OpenData portal (https://opendata.aemet.es; accessed on 12 September 2020) and analyzed with R packages meteoland [25] and geosphere [26]. Considering that the area in the study is dry during the summer and to keep the studied variables at a minimum, the maximum daily temperature (MDT) was selected as a general indicator for heat stress. Other commonly used parameters, such as the temperature–humidity index, provided similar information (e.g., correlation of 0.99), and thus, humidity was not considered relevant, as also found in other studies in Spain [27] and elsewhere [28]. Additionally, the models included the daily light change (DLC; related to increasing or decreasing photoperiod).

In order to identify the periods most relevant for the fertility and prolificacy of the sows regarding MDT and DLC, sows were grouped and averaged for the following periods: 50 to 21 days before AI (possible effect on sperm production), 21 to 14 days before AI (prior effect on sows’ readiness) [28], and 37 days after the AI (fertilization and implantation).

### 2.3. Data Analysis

Data analysis was performed in the R statistical environment v. 4.0.4 [29]. Fertility (AI resulting in farrowing) and piglet counts were analyzed by generalized linear mixed-effects models (GLME; logistic regression for fertility and Poisson for counts) [30]. The fixed effects of the models included season, parity group, and their interaction, and the random effects included the year, sow, and boar.

The seasonal pattern for each treatment was further studied by fitting the data to cosinor models [31]. Cosinor is a robust statistical method used in chronobiology for rhythmicity detection and analysis using cosine curve fitting. It yields a series of parameters for describing the fitted model, as the MESOR (Midline Statistic of Rhythm, a rhythm-adjusted mean), amplitude (amount of variation within each cycle), and acrophase (time to the maximum value of the cycle) of the curve. It is possible to add covariates for comparing periodograms. In our analysis, we included the parity group as a covariate for evaluating its influence on the model. The 2–4 group was used as the reference in all cases, testing if the effect of the parity on the MESOR, amplitude, and acrophase of the parity were significantly different from 0.

We further analyzed the effects of meteorological variables on fertility and parity. Generalized linear mixed-effects models (GLME) were applied, similar to in the season × parity analysis, with the maximum temperature, heat loads, and photoperiod change as explanatory variables and the parity. The analysis was performed for each of the periods for averaging the meteorological variables, as described in Section 2.2.

The results are presented as means ± SE unless otherwise stated. The effects were considered significant at *p* ≤ 0.05, adjusting *p* values in multiple comparisons with Tukey’s method.

## 3. Results

### 3.1. Effects of the Parity and Season on Fertility and Prolificacy of Iberian Sows

**Figure 1:**
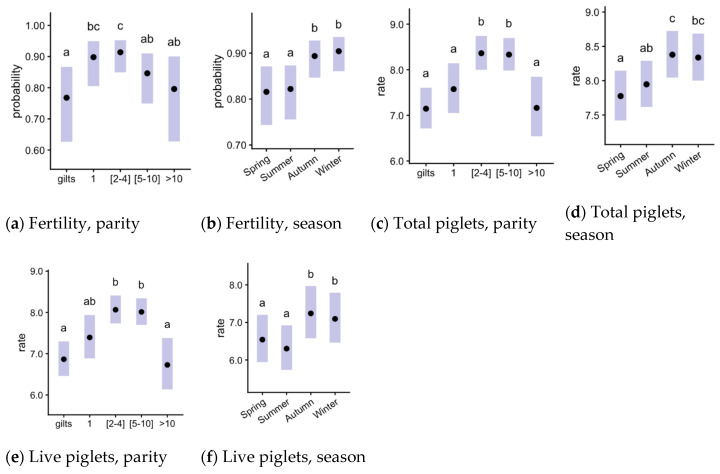
Effects of parity and season in pig fertility and prolificacy for total and live piglets born in gilts and sows with parity 1, 2–4, 5–10 and >10 during summer, spring, autumn and winter periods. The interaction between factors was not significant, so they are shown as the main effects. The plots show estimated means (points) and their 95% confidence intervals (bars). ^a,b,c^ *p* ≤ 0.05 among groups.

Overall (mean ± SD), the average fertility (farrowing rate) was 85.0% ± 0.6, and the prolificacy variables were 8.3 ± 2.3 total piglets per farrowing, 7.9 ± 2.2 born alive, 0.2 ± 0.5 stillborn piglets, and 0.1 ± 0.4 mummified piglets. The season and parity did not interact significantly for fertility, and total piglets born and piglets born alive (Figure 1; Table 1). All of these variables (Figure 1b,d,f) showed significantly higher averages in autumn and winter when compared with spring and summer (summer–winter, *p* > 0.05 for born alive, Figure 1d). Comparing parity groups, sows with 1 and 2–4 farrowings presented the highest farrowing rates (Figure 1a), with 2–4 significantly different from the others. Parities 2–4 and 5–10 presented the highest total born and those born alive (*p* < 0.05 compared with the other groups, except group 1 for born alive).

**Figure 2:**
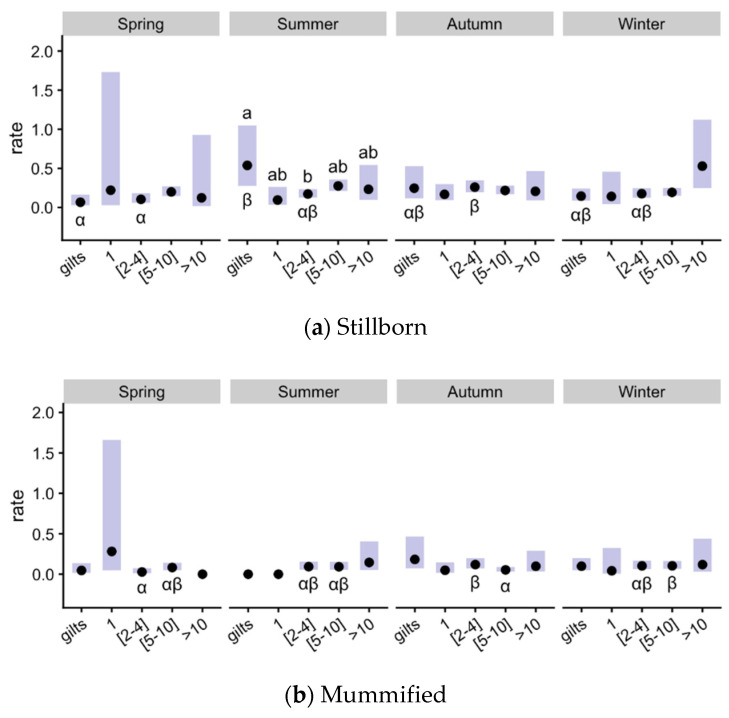
Effects of parity and season on pig prolificacy for stillborn and mummified piglets (significant interaction) in gilts and sows with parity 1, 2–4, 5–10 and >10 during summer, spring, autumn and winter periods. The plots show estimated means (points) and 95% confidence intervals (bars) for each treatment × season combination. ^a,b^ *p* ≤ 0.05 among parity groups within each season, ^αβ^ *p* ≤ 0.05 among season groups within each parity group.

There was a significant interaction of season × parity for stillborn and mummified piglets (Table 1). In this case (Figure 2a), spring presented lower values for gilts (*p* < 0.05 with summer) and parities 2–4 (*p* < 0.05 with autumn). Parity significantly affected the number of stillborn piglets in summer, with gilts presenting the highest values (*p* < 0.05 with parities 2–4, other group intermediates). The number of mummified piglets was only affected by season in parity groups 2–4 and 5–10 (Figure 2b). Parities 2–4 yielded the highest values in autumn (*p* < 0.05 with the lowest in spring) and 5–10 in winter (*p* < 0.05 with autumn).

### 3.2. Fitting Fertility and Prolificacy Results Using Cosinor Models and Comparison among Parity Groups

**Figure 3:**
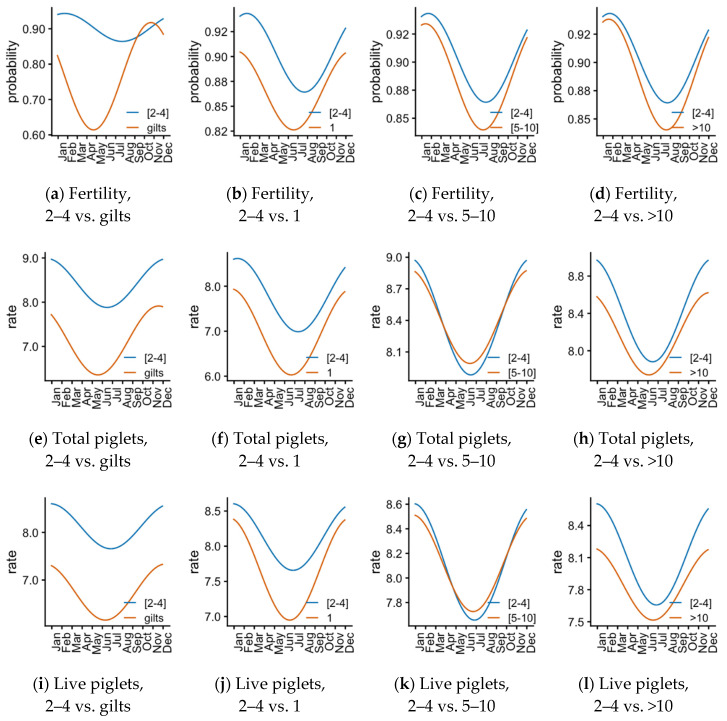

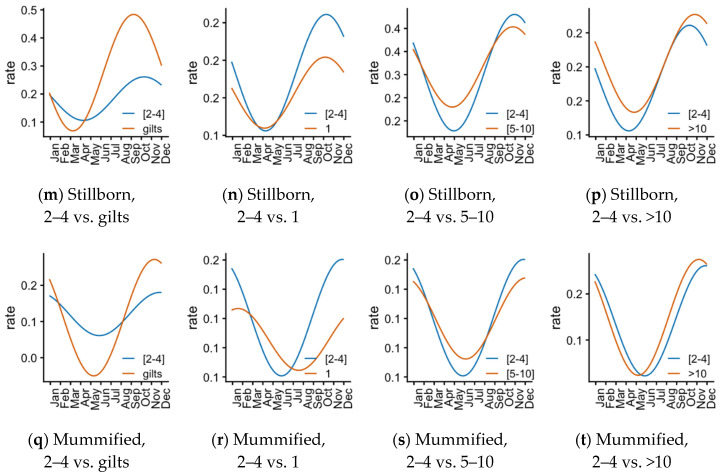
Representation of the seasonal effects after fitting cosinor models in gilts and sows with parity 1, 2–4, 5–10 and >10 between January and December. Each row shows the plots resulting from comparing the reference parity group 2–4 (blue) with each of the others (red). Table 2 shows the statistical significance of the parameters for each model.

The data were fitted to cosinor models [32] to detail the variables’ dynamics during the year. The models (taking parities 2–4 as the reference parity group) are depicted in Figure 3, and the model parameter’s significances are shown in Table 2 (models detailed in the Supplementary Materials) for MESOR (midline estimating statistic of rhythm and intercept for the model), amplitude (half the extent of predictable variation within a cycle) and acrophase (time of high values recurring in each cycle). In most cases, the MESOR for each parity group was significantly different from the reference parities 2–4 (Table 2). Notably, the MESOR for 5–10 was not significantly different for total and live piglets, and no cases were significantly different for mummified piglets. The comparison among the parity groups and reference (“test” columns in Table 2) showed few significant differences. Whereas the acrophase and amplitude were not significantly different in most cases (evident as the similarity of the sinusoids in Figure 3), we detected a high signification for both parameters 2–4 and gilts for fertility. Figure 3a shows how that difference (parameter estimates in Tables S1–S20) reflects the yearly dynamics of farrowings. The amplitude was also significantly different for the gilts group for stillborn and mummified piglets and for parity 5–10 for total, live, and mummified piglets.

### 3.3. Effects of Environmental Variables on Duroc × Iberian Pig Fertility and Prolificacy

To better clarify the effects of seasonality on the reproductive results, we used the maximum daily temperature (MDT) and the day length change (DLC; photoperiod variation) collected at different moments before and after the AI. The parity was significant for all of the studied parameters except for mummified piglets, following the trends found in the season analysis (Table 3, Table 4 and Table 5). Table 3 shows the model significations for the period potentially affecting sperm quality (effect on boars). Both MDT and the DLC negatively influenced farrowing rate, total born, and born alive, but only MDT (positively) affected the number of stillborn piglets. An interaction between parity and DLC was evident only for total born due to a nonsignificant effect of DLC on the 5–10 parity group and, for mummified piglets, showing a strong negative effect of DLC only for gilts, becoming positive for the 2–4 group (*p* < 0.001).

Table 4 shows the model significances for the period affecting sow readiness for AI. In this case, the MDT significantly affected the farrowing rate and total, live, and stillborn piglets (negative relationship), whereas the DLC was significant for all of the fertility variables. However, we found a significant interaction with parity for total and live farrowed and mummified piglets. Examining these interactions, the MDT effect on total and live piglets was not significant for gilts and the 5–10 group, but it was significant (negative relationship) for the parity 1 and 2–4 groups. For mummified piglets, MDT was significant (negative relationship) for the gilts and 2–4 groups and not for the others. There were significant interactions between DLC and parity for stillborn and mummified piglets. DLC showed a significant (negative) effect only in the 2–4 group for stillborn piglets and both in the gilts and 2–4 groups for mummified piglets.

Table 5 shows the model significances for the period immediately after AI. The MDT results were similar to those found in the analysis of the period affecting spermatogenesis but with significant interactions with parity for total farrowed and mummified piglets. In these cases, we found that the MDT effect on total farrowed was not significant for the 5–10 group, and for mummified piglets, similar to the pre-AI analysis, MDT was significant (negative relationship) only for the gilts and 2–4 groups. Interestingly, DLC was only significant for the number of stillborn piglets (negative relationship), showing a significant interaction with parity for the number of mummified piglets. This interaction showed that DLC had a significant negative effect for gilts and a positive one for the 5–10 group.

## 4. Discussion

The effect of season on sow farms’ productivity and reproductive performance is still a challenge for modern industry. Seasonal infertility remains a problem for pig breeding, and studying it is difficult due to many confounding factors. Whereas most problems are due to heat stress affecting the sows during ovulation or pregnancy [33,34], other factors are involved. Seasonal effects are more evident with extensively or semi-extensively reared animals, tropical climates, farms with limited control of the environment, or local breeds with low genetic selection [35]. Other environmental effects such as the photoperiod or an effect on semen quality could be highly relevant, at least in some cases [19,36,37].

A seasonal effect was not detected in previous studies in which AI was performed using the semen supplement Suinfort^®^ in commercial breeds (Landrace and Large White boars, and Landrace × Large White sows) [23]. However, another study on Gochu Asturcelta (a local breed extensively reared) [19] and on Iberian × Duroc [38] reported a significant influence of the period of the year on semen freezability and fertility performance, respectively. The variability among studies highlights the need for considering individual situations. Breeds, environment (latitude and climate), and farming procedures could cause variability between large-scale studies performed with commercial breeds and modern facilities. The present study focused on the Iberian pig [1], a breed raised for its organoleptic and nutritional qualities. Iberian sows have not been subjected to genetic selection, are well adapted to the hot and dry Iberian climate (Southern/inland), and might show some physiological differences from typical commercial breeds.

Whereas the Iberian × Duroc crossing is increasingly popular due to its possibilities for increasing productivity and the quality of the offspring, the bibliography is still scarce [2,38,39]. Here, we demonstrate a critical role of season and parity in the productive performance of Iberian sows. These results are not surprising since parity is known to impact sow fertility [11,12,13]. First, gilts show reduced reproductive performance, as clearly evidenced in our study for fertility and prolificacy. Moreover, consecutive pregnancies affect the sow’s physiology, leading to decreasing reproductive performance, although this depends on individual, breed, and rearing conditions [13,40,41]. Whereas the physiological mechanisms behind the effects of the age of the sow and parity on fertility and prolificacy are still little known [11], it is undeniable that both lower-parity and old sows present suboptimal performance [17,42], and the results on the Iberian × Duroc crossing in our study support those previous findings, with the most consistent and highest fertility and prolificacy results in the parity 2–4 group.

Nevertheless, from the fifth farrowing (parity 5 and at up to 10), sows showed acceptable reproductive performances at the expense of higher variability. The case for primiparous sows was mixed, with fertility comparable with the reference group but having lower prolificacy. These sows could be at a higher risk of miscarriage [28], resulting in fewer farrowed piglets.

In a first approach for analyzing the seasonal variability of the reproductive performance of the Iberian × Duroc breeding, we analyzed the reproductive data within each natural season. Seasonal effects are apparent in tropical or subtropical climates, especially when high humidity combines with heat [11,20,28,43]. However, the climate also affects farm performance in temperate climates, with a decrease mainly observed in the summer and early autumn, and both with liquid-stored [13,22,27,28,44,45,46] and cryopreserved semen [47]. In a recent study on Spanish herds [48], the miscarriage occurrence was higher in the summer and early autumn. These effects are related to insufficient thermoregulation in this species [27], impacting ovarian function [33,49]. However, other effects such as the photoperiod could also be relevant, although potentially minor in farms having photoperiod-controlled facilities [7]. Our results agree with our preliminary report on Duroc × Iberian [38] and differ from previous studies in swine (with a decrease in fertility in summer and the early autumn) by presenting a clear distinction between the spring–summer and autumn–winter periods.

Interestingly, neither farrowing rate nor total/live piglets at farrowing showed evidence of interactions between parity and season and, therefore, our results could not be attributed to a skewed distribution due to parity groups. A hypothesis is that early miscarriages could have a role in skewing our data towards lower results (both fertility and piglets born) earlier in the year. It is important to consider that the sows were Iberian, a breed still little researched regarding the reproductive parameters and physiological peculiarities [50], and possibly with lower reproductive performance.

Moreover, the use of Duroc semen could influence our results, as these boars show lower fertility than other commercial breeds [51]. Interestingly, we obtained an inverse distribution when studying the post-thawing semen quality of another autochthonous Spanish breed, the Gochu Asturcelta [19], with better post-thawing quality when the semen had been collected in spring and decreasing towards the winter. While the experimental conditions are not comparable (liquid vs. cryopreserved storage), these observations support the need for further research on the reproductive characteristics of local breeds for better management. However, the possible environmental influence on sperm fertility must be cautiously considered since we could not study sperm quality in detail. This aspect remains to be further investigated in future studies.

Since the interpretation of our results is complex due to the interaction of many factors in seasonal infertility (breed, local climate, and farm management), we further explored the data to assess the importance of the environmental variables on the reproductive performance of Iberian sows. Cosinor models, to our knowledge, have not previously been used to analyze reproductive performance in farm animals. The periodograms produced from the cosinor models reflected the trend found by the seasonal analysis, with the reproductive parameters for farrowing rate and farrowed piglets dropping by spring and reaching a minimum by summer but recovering between summer and autumn. Moreover, the cosinor analysis allowed for the detection of some striking differences between our reference group (parities 2–4) and the gilts. The fertility and prolificacy of different parity groups were the lowest by summer, but this effect was shown earlier for gilts, especially for pregnancy. Moreover, the variation in stillborn piglets was higher than in other groups and presented the minimum and maximum incidences earlier in the year (end of winter and summer, respectively). Gilts are more sensitive to heat stress or photoperiod variations regarding their reproductive readiness [52], partly explaining our results.

A limitation of this study is that some data, such as sow age or pre-farrowing removal, was not available in the farm’s dataset. The age at AI could be a relevant factor for reproductive success, especially for gilts, and could affect many levels. For instance, age at first AI has been related to gilt fertility and the lifetime performance of sows [8]. Moreover, gilts could be more sensitive to heat stress, including peripartum death [53]. In this study, the maximum temperature and the photoperiod change were the environmental parameters of choice, following previous reports [54], and breed could modulate their impact on reproductive performance [13,41]. Both parameters were indeed significant in our analyses.

Although animals housed in modern farms are partially insulated from the natural environment, hot weather and changing photoperiod still affect them. Here, we confirmed a role for both the ambient temperature and the photoperiod change, helping to explain our findings when comparing seasons and the yearly rhythms. Pigs are sensitive to heat stress [53], affecting spermatogenesis and the capacity of the sows to prepare for and maintain a pregnancy [27,33,55]. Interestingly, whereas heat influenced fertility and the number of piglets farrowed in the three periods studied, the sows seemed not to be affected by the photoperiod change during the period following AI. MDT could impact the optimal 2–4 parity group more, considering the interactions from our models. Therefore, although Iberian pigs could be more adapted to the warm–hot climate of the farm’s environment, farms might improve sow performance if animals remain in their thermoneutral zone [7]. This should be a priority in the face of more frequent heat waves due to climate change [56]. However, the increasing photoperiod could play a critical role during the spring and even summer [54,57,58].

## 5. Conclusions

Iberian sows bred with Duroc semen present a clear seasonality, differing from previous studies in that they present lower performance results earlier in the year. Parity also has a relevant effect on reproductive performance, being optimal between two to four farrowings. The information provided from this analysis and the models extracted from the cosinor analysis could improve the management of this valuable breed.

## Figures and Tables

**Table 1:** *p*-values for the fixed part of the linear mixed models to analyze the role of parity and season effects on the fertility and prolificacy variables.

Variable	Parity	Season	Parity × Season
Pregnant	<0.001	<0.001	0.720
Total born	<0.001	<0.001	0.205
Live born	<0.001	0.001	0.312
Stillborn	0.090	0.022	0.006
Mummified	0.853	0.643	0.049

**Table 2:** *p*-values for the cosinor models’ parameters (Figure 3). In all cases, the reference group was 2–4. The MESOR *p*-values for this level correspond to the test H_0_ = 0, whereas the other levels show if the difference with 2–4 is different from 0. The *p*-values for amplitude and acrophase refer to H_0_ = 0, while *p*-values for the test on amplitude and acrophase indicate a significant difference with 2–4 for these parameters. Complete tables with estimated parameters are shown in the Supplementary Materials.

Variable	Parity	MESOR *	Amplitude †	Acrophase ‡	Amplitude Test	Acrophase Test
Fertility ratio	gilts	<0.001	<0.001	0.001	0.003	<0.001
	1	0.035	0.044	0.629	0.997	0.345
	2–4	<0.001	0.006	0.036	-	-
	5–10	<0.001	<0.001	0.009	0.142	0.400
	>10	0.056	<0.001	0.011	0.349	0.821
Total born	gilts	<0.001	0.003	0.470	0.423	0.214
	1	<0.001	<0.001	0.349	0.168	0.953
	2–4	<0.001	<0.001	0.244	-	-
	5–10	0.937	<0.001	0.295	0.002	0.395
	>10	<0.001	<0.001	0.853	0.179	0.266
Born alive	gilts	<0.001	0.017	0.707	0.663	0.538
	1	<0.001	<0.001	0.282	0.087	0.546
	2–4	<0.001	<0.001	0.067	-	-
	5–10	0.661	<0.001	0.084	0.014	0.238
	>10	<0.001	0.002	0.417	0.077	0.449
Stillborn	gilts	0.052	<0.001	<0.001	0.031	0.186
	1	0.400	0.265	0.173	0.476	0.934
	2–4	<0.001	0.003	0.003	-	-
	5–10	0.029	0.004	<0.001	0.088	0.091
	>10	0.111	0.012	0.079	0.778	0.285
Mummified	gilts	0.793	<0.001	0.177	0.045	0.500
	1	0.381	0.243	0.449	0.357	0.395
	2–4	<0.001	0.004	0.801	-	-
	5–10	0.867	0.007	0.870	0.011	0.192
	>10	0.737	0.007	0.209	0.853	0.197

* MESOR (midline estimating statistic of rhythm). † Amplitude (half the extent of predictable variation within a cycle). ‡ Acrophase (time of high values recurring in each cycle).

**Table 3:** Effects of parity and environmental variables during 50 to 21 d before AI (estimate for environment effects on semen quality) on pig fertility and prolificacy.

Fertility Variable	Parity Effect	MDT ^1^ Effect	DLC ^2^ Effect	Parity × MDT	Parity × DLC
Pregnant	<0.001	<0.001	<0.001	0.233	0.971
Total born	<0.001	0.003	<0.001	0.054	0.041
Live born	<0.001	<0.001	<0.001	0.086	0.126
Stillborn	0.049	0.003	0.310	0.301	0.072
Mummified	0.134	0.139	0.111	0.666	<0.001

^1^ Maximum daily temperature. ^2^ DLC: day length change, the day length difference between consecutive days (photoperiodic variation).

**Table 4:** Effects of parity and environmental variables 21 to 14 d before AI (estimate for environment effects on the sow, pre-AI) on pig fertility and prolificacy.

Fertility Variable	Parity Effect	MDT ^1^ Effect	DLC ^2^ Effect	Parity × MDT	Parity × DLC
Pregnant	<0.001	<0.001	<0.001	0.674	0.619
Total born	<0.001	<0.001	<0.001	0.016	0.154
Live born	<0.001	<0.001	0.003	0.049	0.429
Stillborn	0.048	0.068	0.028	0.225	0.043
Mummified	0.159	0.059	0.216	0.019	<0.001

^1^ Maximum daily temperature. ^2^ DLC: day length change, the day length difference between consecutive days (photoperiodic variation).

**Table 5:** Effects of parity and environmental variables 1 to 37 d after AI (estimate for environment effects on the sow, post-AI) on pig fertility and prolificacy.

Fertility Variable	Parity Effect	MDT ^1^ Effect	DLC ^2^ Effect	Parity × MDT	Parity × DLC
Pregnant	<0.001	<0.001	0.413	0.641	0.057
Total born	<0.001	<0.001	0.304	0.036	0.263
Live born	<0.001	<0.001	0.621	0.157	0.525
Stillborn	0.049	0.116	<0.001	0.112	0.068
Mummified	0.152	0.281	0.731	<0.001	<0.001

^1^ Maximum daily temperature. ^2^ DLC: day length change, the day length difference between consecutive days (photoperiodic variation).

## Data Availability

Data can be sent to readers upon request.

## Supplementary Materials

The following are available online at https://www.mdpi.com/article/10.3390/ani11113275/s1, Table S1–S20: Supplementary tables for the cosinor models. The MESOR is the midline estimating statistic of rhythm; the amplitude is half the extent of predicted variation within a cycle; and the acrophase is the time of high values recurring in each cycle. In all models, the [2,3,4] parity group was used as the reference.
