# Supplementary material for: Effect of Season and Parity on Reproduction Performance of Iberian Sows Bred with Duroc Semen
Source: Animals (Basel). 2021 Nov 16;11(11):3275. doi: 10.3390/ani11113275 (PMC8614353; doi:10.3390/ani11113275)
Supplement: Supplementary file 1 [file animals-11-03275-s001.zip › animals-1440977-supplementary.pdf]

animals

Effect of season and parity on reproduction performance of Iberian sows bred with Duroc semen.

Javier Piñán [1], [2], Beatriz Alegre [1,2], Roy N. Kirkwood [3], Cristina Soriano-Úbeda [1], Magdalena Maj [4], Juan Carlos Domínguez [1,2], Rodrigo Manjarín[5,*] and Felipe Martínez-Pastor [1,6]

[1] Institute of Animal Health and Cattle Development (INDEGSAL), Universidad de León, 24071 León, Spain
[2] Animal Science Department, California Polytechnic State University, One Grand Ave, Bldg. 10, San Luis Obispo, CA, 93407-0255
[3] School of Animal and Veterinary Sciences, University of Adelaide, Roseworthy, SA, Australia, SA 5371
[4] Department of Animal Medicine, Surgery and Anatomy (Animal Medicine and Surgery), Universidad de León, 24071 León, Spain
[5] Department of Molecular Biology (Cell Biology), Universidad de León, 24071 León, Spain
* Correspondence: rmanjari@calpoly.edu; Tel.: +1 (805) 756-7474

Supplementary tables for the cosinor models. The MESOR is the midline estimating statistic of rhythm; the amplitude is half the extent of predicted variation within a cycle; and the acrophase is the time of high values recurring in each cycle. In all models, the [2–4] parity group was used as the reference.

Table S1. Fertility: Parameters for the cosinor model fitted with parity groups [2–4] (as reference) and gilts.

Parameter	Estimate	SE	Lower CI	Upper CI	P value
MESOR [2–4]	0.904	0.011	0.882	0.926	<0.001
MESOR gilts	-0.138	0.028	-0.194	-0.082	<0.001
Amplitude	0.040	0.015	0.011	0.068	0.006
Amplitude:gilts	0.152	0.035	0.084	0.219	<0.001
Acrophase	0.878	0.420	0.055	1.701	0.036
Acrophase:gilts	-0.689	0.217	-1.114	-0.264	0.001

Table S2. Fertility: Parameters for the cosinor model fitted with parity groups [2–4] (as reference) and 1.

Parameter	Estimate	SE	Lower CI	Upper CI	P value
MESOR [2–4]	0.904	0.010	0.883	0.924	<0.001
MESOR 1	-0.038	0.018	-0.073	-0.003	0.035
Amplitude	0.040	0.014	0.013	0.066	0.004
Amplitude:1	0.040	0.020	0.001	0.078	0.044
Acrophase	0.878	0.395	0.105	1.652	0.026
Acrophase:1	0.290	0.601	-0.888	1.468	0.629

Table S3. Fertility: Parameters for the cosinor model fitted with parity groups [2–4] (as reference) and [5–10].

Parameter	Estimate	SE	Lower CI	Upper CI	P value
MESOR [2–4]	0.904	0.011	0.881	0.926	<0.001
MESOR [5–10]	-0.017	0.004	-0.024	-0.010	<0.001
Amplitude	0.040	0.015	0.010	0.069	0.008
Amplitude:[5–10]	0.047	0.012	0.025	0.070	<0.001
Acrophase	0.878	0.429	0.037	1.720	0.041
Acrophase:[5–10]	0.726	0.277	0.184	1.269	0.009

Table S4. Fertility: Parameters for the cosinor model fitted with parity groups [2–4] (as reference) and >10.

Parameter	Estimate	SE	Lower CI	Upper CI	P value
MESOR [2–4]	0.904	0.010	0.883	0.924	<0.001
MESOR >10	-0.015	0.008	-0.029	0.000	0.056
Amplitude	0.040	0.014	0.013	0.066	0.004
Amplitude:>10	0.049	0.015	0.020	0.077	<0.001
Acrophase	0.878	0.392	0.110	1.647	0.025
Acrophase:>10	0.823	0.324	0.187	1.458	0.011

Table S5. Total piglets born: Parameters for the cosinor model fitted with parity groups [2–4] (as reference) and gilts.

Parameter	Estimate	SE	Lower CI	Upper CI	P value
MESOR [2–4]	7.802	0.106	7.594	8.011	<0.001
MESOR gilts	-1.629	0.271	-2.160	-1.098	<0.001
Amplitude	0.814	0.139	0.542	1.087	<0.001
Amplitude:gilts	1.225	0.327	0.584	1.867	<0.001
Acrophase	0.701	0.193	0.322	1.080	<0.001
Acrophase:gilts	0.167	0.257	-0.338	0.672	0.517

Table S6. Total piglets born: Parameters for the cosinor model fitted with parity groups [2–4] (as reference) and 1.

Parameter	Estimate	SE	Lower CI	Upper CI	P value
MESOR [2–4]	7.802	0.108	7.590	8.014	<0.001
MESOR 1	-0.815	0.185	-1.178	-0.452	<0.001
Amplitude	0.814	0.141	0.538	1.091	<0.001
Amplitude:1	0.958	0.200	0.565	1.350	<0.001
Acrophase	0.701	0.196	0.316	1.085	<0.001
Acrophase:1	0.346	0.257	-0.158	0.850	0.179

Table S7. Total piglets born: Parameters for the cosinor model fitted with parity groups [2–4] (as reference) and [5–10].

Parameter	Estimate	SE	Lower CI	Upper CI	P value
MESOR [2–4]	7.802	0.111	7.584	8.020	<0.001
MESOR [5–10]	-0.095	0.035	-0.164	-0.026	0.007
Amplitude	0.814	0.145	0.530	1.099	<0.001
Amplitude:[5–10]	0.783	0.113	0.562	1.005	<0.001
Acrophase	0.701	0.202	0.305	1.097	<0.001
Acrophase:[5–10]	0.688	0.162	0.370	1.007	<0.001

Table S8. Total piglets born: Parameters for the cosinor model fitted with parity groups [2–4] (as reference) and >10.

Parameter	Estimate	SE	Lower CI	Upper CI	P value
MESOR [2–4]	7.802	0.107	7.592	8.013	<0.001
MESOR >10	-0.273	0.078	-0.427	-0.120	<0.001
Amplitude	0.814	0.140	0.539	1.089	<0.001
Amplitude:>10	0.750	0.151	0.455	1.045	<0.001
Acrophase	0.701	0.195	0.318	1.084	<0.001
Acrophase:>10	0.623	0.217	0.197	1.048	0.004

Table S9. Live piglets born: Parameters for the cosinor model fitted with parity groups [2–4] (as reference) and gilts.

Parameter	Estimate	SE	Lower CI	Upper CI	P value
MESOR [2–4]	7.521	0.102	7.322	7.721	<0.001
MESOR gilts	-1.682	0.259	-2.189	-1.174	<0.001
Amplitude	0.771	0.132	0.512	1.030	<0.001
Amplitude:gilts	1.108	0.309	0.503	1.713	<0.001
Acrophase	0.833	0.196	0.449	1.217	<0.001
Acrophase:gilts	0.391	0.276	-0.149	0.932	0.156

Table S10. Live piglets born: Parameters for the cosinor model fitted with parity groups [2–4] (as reference) and 1.

Parameter	Estimate	SE	Lower CI	Upper CI	P value
MESOR [2–4]	7.521	0.103	7.319	7.724	<0.001
MESOR 1	-0.760	0.177	-1.108	-0.413	<0.001
Amplitude	0.771	0.134	0.508	1.034	<0.001
Amplitude:1	0.912	0.191	0.538	1.286	<0.001
Acrophase	0.833	0.199	0.443	1.223	<0.001
Acrophase:1	0.375	0.259	-0.133	0.882	0.148

Table S11. Live piglets born: Parameters for the cosinor model fitted with parity groups [2–4] (as reference) and [5–10].

Parameter	Estimate	SE	Lower CI	Upper CI	P value
MESOR [2–4]	7.521	0.106	7.313	7.730	<0.001
MESOR [5–10]	-0.102	0.034	-0.168	-0.036	0.002
Amplitude	0.771	0.138	0.500	1.042	<0.001
Amplitude:[5–10]	0.750	0.108	0.539	0.961	<0.001
Acrophase	0.833	0.205	0.431	1.235	<0.001
Acrophase:[5–10]	0.782	0.163	0.463	1.102	<0.001

Table S12. Live piglets born: Parameters for the cosinor model fitted with parity groups [2–4] (as reference) and >10.

Parameter	Estimate	SE	Lower CI	Upper CI	P value
MESOR [2–4]	7.521	0.102	7.321	7.721	<0.001
MESOR >10	-0.295	0.074	-0.441	-0.150	<0.001
Amplitude	0.771	0.133	0.511	1.031	<0.001
Amplitude:>10	0.691	0.142	0.412	0.970	<0.001
Acrophase	0.833	0.197	0.448	1.219	<0.001
Acrophase:>10	0.772	0.225	0.331	1.212	<0.001

Table S13. Stillborn piglets: Parameters for the cosinor model fitted with parity groups [2–4] (as reference) and gilts.

Parameter	Estimate	SE	Lower CI	Upper CI	P value
MESOR [2–4]	0.184	0.018	0.149	0.218	<0.001
MESOR gilts	0.093	0.048	-0.001	0.186	0.052
Amplitude	0.078	0.026	0.026	0.129	0.003
Amplitude:gilts	0.208	0.054	0.101	0.314	<0.001
Acrophase	-0.908	0.301	-1.498	-0.317	0.003
Acrophase:gilts	-1.450	0.278	-1.994	-0.906	<0.001

Table S14. Stillborn piglets: Parameters for the cosinor model fitted with parity groups [2–4] (as reference) and 1.

Parameter	Estimate	SE	Lower CI	Upper CI	P value
MESOR [2–4]	0.184	0.017	0.149	0.218	<0.001
MESOR 1	-0.027	0.032	-0.089	0.035	0.4
Amplitude	0.078	0.026	0.027	0.128	0.003
Amplitude:1	0.047	0.042	-0.036	0.130	0.265
Acrophase	-0.908	0.296	-1.488	-0.327	0.002
Acrophase:1	-0.963	0.707	-2.349	0.423	0.173

Table S15. Stillborn piglets: Parameters for the cosinor model fitted with parity groups [2–4] (as reference) and [5–10].

Parameter	Estimate	SE	Lower CI	Upper CI	P value
MESOR [2–4]	0.184	0.019	0.147	0.220	<0.001
MESOR [5–10]	0.013	0.006	0.001	0.025	0.029
Amplitude	0.078	0.028	0.023	0.132	0.005
Amplitude:[5–10]	0.061	0.021	0.019	0.103	0.004
Acrophase	-0.908	0.316	-1.527	-0.288	0.004
Acrophase:[5–10]	-1.089	0.318	-1.712	-0.465	<0.001

Table S16. Stillborn piglets: Parameters for the cosinor model fitted with parity groups [2–4] (as reference) and >10.

Parameter	Estimate	SE	Lower CI	Upper CI	P value
MESOR [2–4]	0.184	0.019	0.147	0.220	<0.001
MESOR >10	0.022	0.014	-0.005	0.048	0.111
Amplitude	0.078	0.027	0.024	0.131	0.005
Amplitude:>10	0.072	0.029	0.016	0.128	0.012
Acrophase	-0.908	0.314	-1.522	-0.293	0.004
Acrophase:>10	-0.630	0.359	-1.335	0.074	0.079

Table S17. Mummified piglets: Parameters for the cosinor model fitted with parity groups [2–4] (as reference) and gilts.

Parameter	Estimate	SE	Lower CI	Upper CI	P value
MESOR [2–4]	0.121	0.014	0.093	0.149	<0.001
MESOR gilts	-0.010	0.039	-0.086	0.066	0.793
Amplitude	0.060	0.021	0.019	0.100	0.004
Amplitude:gilts	0.161	0.046	0.070	0.251	<0.001
Acrophase	-0.083	0.330	-0.729	0.563	0.801
Acrophase:gilts	-0.373	0.276	-0.915	0.169	0.177

Table S18. Mummified piglets: Parameters for the cosinor model fitted with parity groups [2–4] (as reference) and 1.

Parameter	Estimate	SE	Lower CI	Upper CI	P value
MESOR [2–4]	0.121	0.014	0.093	0.148	<0.001
MESOR 1	-0.022	0.025	-0.072	0.028	0.381
Amplitude	0.060	0.020	0.020	0.099	0.003
Amplitude:1	0.032	0.027	-0.022	0.085	0.243
Acrophase	-0.083	0.322	-0.713	0.547	0.796
Acrophase:1	0.809	1.067	-1.284	2.901	0.449

Table S19. Mummified piglets: Parameters for the cosinor model fitted with parity groups [2–4] (as reference) and [5–10].

Parameter	Estimate	SE	Lower CI	Upper CI	P value
MESOR [2–4]	0.121	0.014	0.093	0.148	<0.001
MESOR [5–10]	-0.001	0.005	-0.010	0.008	0.867
Amplitude	0.060	0.020	0.020	0.099	0.003
Amplitude:[5–10]	0.042	0.015	0.011	0.072	0.007
Acrophase	-0.083	0.323	-0.715	0.550	0.797
Acrophase:[5–10]	0.060	0.366	-0.657	0.777	0.87

Table S20. Mummified piglets: Parameters for the cosinor model fitted with parity groups [2–4] (as reference) and >10.

Parameter	Estimate	SE	Lower CI	Upper CI	P value
MESOR [2–4]	0.121	0.015	0.091	0.150	<0.001
MESOR >10	0.004	0.011	-0.018	0.025	0.737
Amplitude	0.060	0.022	0.017	0.102	0.006
Amplitude:>10	0.063	0.023	0.017	0.109	0.007
Acrophase	-0.083	0.347	-0.763	0.597	0.811
Acrophase:>10	-0.424	0.337	-1.085	0.237	0.209
